# Assessing Lifespan and Aging Phenotypes Resulting From FOXO3 Induction Using Mouse Models

**DOI:** 10.1093/geroni/igab046.1417

**Published:** 2021-12-17

**Authors:** Jesse Owens, Brian Hew, Christopher Tran, Kristal Xie, Bradley Willcox

**Affiliations:** 1 Kuakini Medical Center, Honolulu, Hawaii, United States; 2 Kuakini Medical Center, Kuakini Medical Center/Honolulu, Hawaii, United States

## Abstract

Environmental signals, including caloric restriction and oxidative stress, trigger FoxO3 to upregulate genes involved in stress resistance, metabolism, cell cycle arrest, and apoptosis that may help mitigate age-related diseases. Activation of FoxO3 has been shown to have a profound life-extending effect on model organisms. Protective SNPs in FOXO3 are strongly associated with exceptional longevity in humans. The objective of this study is test the relation between FoxO3 and longevity using mouse models. We generated a mouse line containing an extra copy of FoxO3 that can be induced at any age. In our model, FoxO3 remains driven by its natural promoter to avoid mis-expression in inappropriate cells and to maintain the gene’s ability to respond to signals such as stress. We are utilizing this new model to assess survival endpoints and test a panel of aging phenotypes reflecting healthspan throughout the mouse lifespan and compare these to similar human phenotypes.

